# Living on Site While Renovating; Flexible Instructional Design of Post-Graduate Medical Training

**DOI:** 10.5334/pme.1198

**Published:** 2024-05-13

**Authors:** Peter K. H. Deschamps, Geke M. J. Beugels, J. Dudink, Joost Frenkel, Marije P. Hennus, Marijke B. Hofstra, Alexa X. Rutten, Marieke Van der Schaaf

**Affiliations:** 1 Academic Centre for Child and Adolescent Psychiatry and Educational Researcher Associated with the Radboud Health Academy, Nijmegen, The Netherlands; 2 Academic Centre for Child and Adolescent Psychiatry, Ede, the Netherlands; 3 Wilhelmina Children’s Hospital, University Medical Center Utrecht, Utrecht, the Netherlands; 4 Sophia Children’s Hospital, Erasmus Medical Center, Rotterdam, the Netherlands; 5 GGzE, Centre for Child and Adolescent Psychiatry, Eindhoven, the Netherlands; 6 Utrecht Center for Research and Development of Health Professions Education, University Medical Center Utrecht, The Netherlands

## Abstract

**Background:**

Developing theoretical courses for post-graduate medical training that are aligned to current workplace-based learning practices and adaptive to change in the field is challenging, especially in (sub) specialties where time for re-design is limited and needs to be performed while education continues.

**Approach:**

An instructional design method was applied based on flexible co-design to improve post-graduate theoretical courses in child and adolescent psychiatry (CAP) in the Netherlands. In four phases over a period of three years, courses were re-designed at a national level.

**Evaluation:**

Once common vision and learning goals were agreed upon and the prototype was developed (phases 1 and 2), the first courses could be tested in daily practice (phase 3). Phase 4 refined these courses in brief iterative cycles and allowed for designing additional courses building on and adding to previous experiences in brief iterative cycles. The resulting national theoretical courses re-allocated resources previously spent on a local level using easily accessible online tools. This allowed trainees to align content with their clinical rotations, personal preferences and training schedules.

**Reflection:**

The development of theoretical courses for post-graduate medical training in smaller medical (sub-)specialties with limited resources may profit from a flexible instructional design method. We consider the potential merit of such a method to other medical specialties and other (inter-)national efforts to develop theoretical teaching courses. A longer-term implementation evaluation is needed to show to what extent the investment made in the re-design proves to be future-proof and enables rapid adaptation to changes in the field.

## Background and the need for innovation

If building a house is a metaphor for instructional design of education, most methods would advise aspiring homeowners to acquire land, plan carefully with an architect, select materials and craftsmen, and start building a new house from scratch regardless of their current living situation. In reality, families often find themselves renovating their home while still living on site due to budget constraints. The latter scenario of gradual co-design may very well be a metaphor for the flexible (re)design of courses as part of a postgraduate medical training, during the flow of training programs in dynamic professional fields.

This paper reports a flexible instructional (re)design method for theoretical courses in post graduate medical training, which we applied within the medical sub-specialty of child and adolescent psychiatry (CAP) in the Netherlands.

Recent changes in postgraduate medical training, including CAP [1], demand more personalized and flexible training to cultivate adaptable professionals [2,3]. Evolving evidence on brain development and on the importance of mental health resilience and vulnerability for disorders across the life span needs to be integrated into (child and adolescent) psychiatry training. Societal workforce shifts require accommodating part-time schedules of trainees. Additionally, CAP trainees are encouraged to tailor their learning, fostering a lifelong learning attitude, while postgraduate training converges with continuous medical education, offering, at least in the Netherlands, adjusted tracks for general psychiatrists entering CAP later in their careers [4]. Together, these changes in the field and context of CAP pose challenges to clinical educators to re-design training in CAP, including theoretical courses.

As clinician educators in CAP hold clinical responsibilities in an already overwhelmed mental health care system, they have little time and resources for teaching and re-design of their theoretical courses. Most traditional instructional design (ID) models include five or six steps of design activities that overlap with the overarching acronym ADDIE [5], referring to: analyze, design, develop, implement and evaluate. A common example can be found in Kern et al.’s [6] model for ID including: 1. Problem identification and general needs assessment; 2. Targeted needs assessment; 3. Goals and Objectives; 4. Educational Strategies; 5. Implementation; 6. Evaluation and Feedback. Such an approach provides a structured process for developing comprehensive curricula in medical education, focusing on achieving specific learning objectives through systematic planning and implementation. However, these redesign methods do not always fit clinician educator’s practice. Other ID models are more flexible, iterative, based on co-design with stakeholders and increasingly embedding technology. Such is Salmon et al’s [7,8] Carpe Diem instructional design model, which emphasizes active learning, technology integration, flexibility, collaboration, and continuous feedback. Carpe Diem emphasizes seizing the moment and allows a straightforward and efficient approach for re-design that can be weaved into ongoing training programs.

## Goal of innovation

The dual goal of the innovation was to align the *content* and *form* of theoretical courses with the changing needs in the field of CAP and workplace within the limits of feasibility in the Netherlands.

## Phases taken for development and implementation of innovation

### The stakeholders panel

According to the Carpe Diem method, a small panel of stakeholders, representing the four regions of the Netherlands where CAP theoretical training was offered(n = 10), was brought together, consisting of training program directors/teachers, trainees, an administrative support assistant and an educational specialist. Throughout the re-design process, the panel communicated every phase with the national assembly of CAP training program directors to assure co-construction with a larger stakeholder’s group.

### Carpe Diem design method

**Figure 1:**
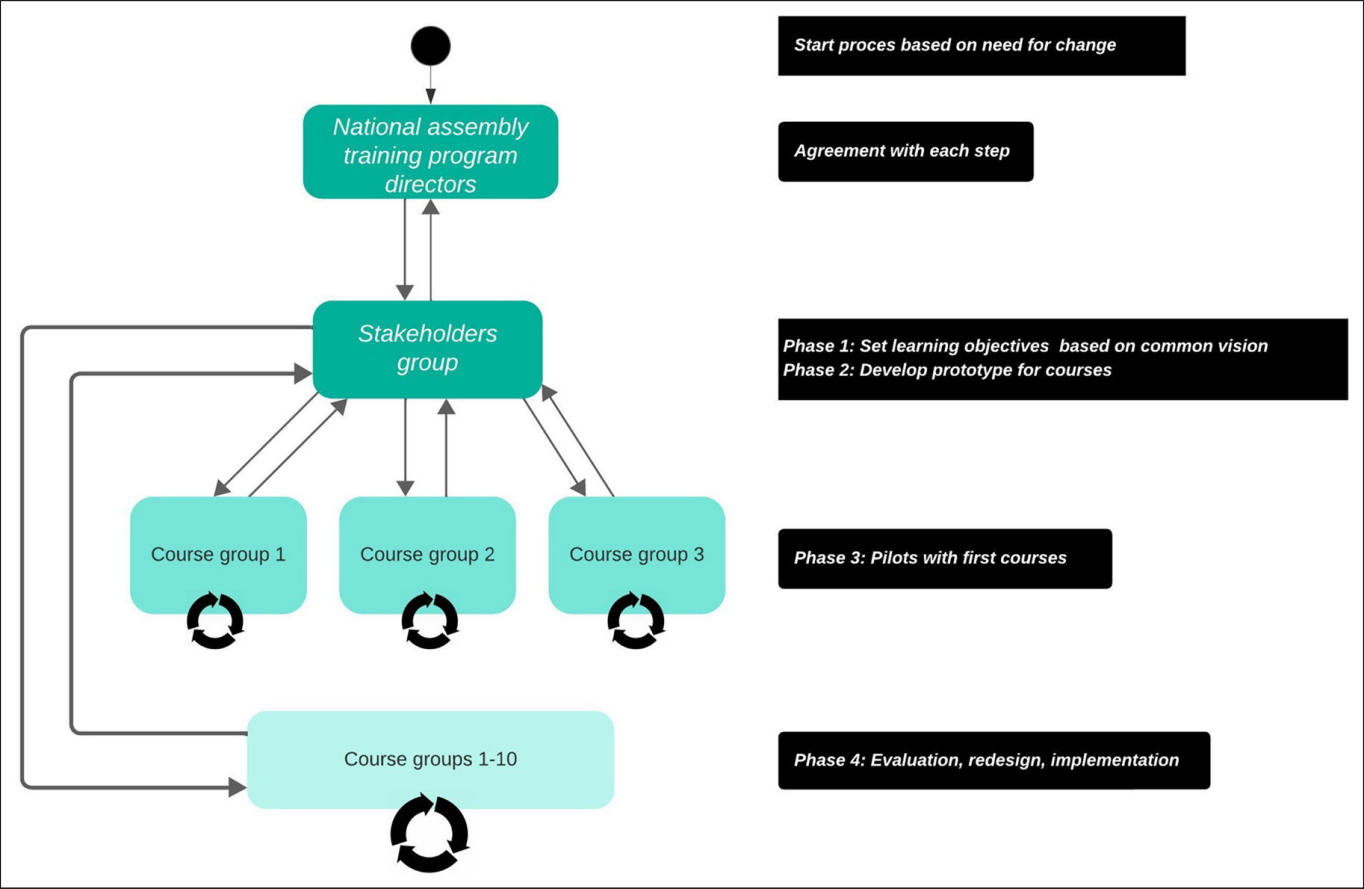
The redesign process: once common vision and learning goals are agreed upon and the prototype is developed (phases 1 and 2), the first courses can be tested in daily practice (phase 3). Phase 4 refines these courses in brief iterative cycles and allows for designing additional courses building on and adding to previous experiences in brief iterative cycles.

This contribution shows a flexible instructional design procedure based on the *Carpe Diem* method [7] that applies principles of flexibility, technology and co-design [9]. Typically, in one rather concise session a common ground of vision (step 1) and learning goals (step 2) are agreed upon by all stakeholders and prototypes are designed that can readily be integrated into daily practice (step 3). The last three steps refine the prototypes in brief iterative cycles with evaluation (step 4), adaptation (step 5) and further implementation (step 6) merging daily practice with re-design. The current project applied these steps in 4 phases as depicted in Figure 1.

### First phase: Agreement on learning objectives based on a common vision

A request for information was sent out to CAP training institutes in the Netherlands requesting their current CAP learning objectives and training practices. Responses were combined in an inventory of learning objectives by a CAP trainee and training program director. The recently updated European Curriculum Framework [10,11] was used as an additional source to add objectives to the inventory. During a first meeting, the stakeholders panel formulated a common vision for future CAP training around the central question: What does a child and adolescent psychiatrist need during training to best prepare them to deliver optimal care for children and their families in the future? With this common vision in mind, items from the inventory of learning objectives were retained, removed, refined, complemented, and ordered using a storyboard. At the end of this session, which lasted approximately six hours, a learning goals inventory was ready to send out to all training program directors in the Netherlands for comments and broader agreement.

### Second phase: Developing a prototype of courses for CAP training

An inventory of teaching methods preferences was made, combining written evaluation forms filled in by trainees and teachers after regional theoretical courses in the prior 2 years with in-depth interviews with 10 CAP trainees (representatives of all four regions). These forms and interviews enquired about the most inspiring and least helpful experiences. During a meeting with the stakeholders panel, based on these teaching methods preferences and the common vision, several guiding principles were defined for the prototypes of course modules (phase 2a). Combined with the learning goals from step 1, a modular program of theoretical courses was proposed (phase 2b). At the end of this session, which lasted approximately four hours, the results were sent out to all training program directors in the Netherlands for comments and agreement.

### Third phase: Pilots of courses

A system with online media walls was introduced as a tool to help organize the development of teaching courses and facilitate student and teacher engagement [12]. These allowed multiple people to work on a project and to share teaching materials easily for redesign as well as for daily teaching practice. Pilots were conducted with three teaching courses, constructed at a national level by a core group including a coordinator, around seven trainers/teachers and one or two trainees. Feedback collected each time a course was provided in a region was used to re-design the next iteration. Ethical approval was not applicable as the evaluations were collected in light of standard practice quality assurance of courses in CAP training in the Netherlands.

### Fourth phase: Evaluation, redesign, and further implementation

Feedback from the first three courses was used to start up new core groups for consecutive courses. This led to full coverage of learning goals in a total of 16 courses. When a course was offered in a region, evaluation was used for further redesign at a national level. This facilitated the implementation and the start of a full cycle across regions with national coordination.

## Outcomes of the innovation

### First phase: Agreement on learning objectives based on a common vision

The vision included two central notions: a developmental mindset in the assessment and treatment of mental health problems in children; and a systems approach, including circular effects of the environment on child mental health, societal and cultural effects. A list of learning objectives was build based on this vision. The summary is listed in Table 1.

**Table 1:** Summary of results from phase 1 *Agreement on learning objectives based on a common vision* and phase 2 *Developing a prototype of courses for CAP training*.


PHASE 1: AGREEMENT ON LEARNING OBJECTIVES BASED ON A COMMON VISION	

**Category**	**Example of learning goal**

General etiology	Knowledge on development of vulnerability and resilience in children

General assessment	Knowledge on stages of typical and abnormal child development.

General treatment	Specific CAP treatment skills, including creating working alliance with children and parents/care givers.

Specific conditions	Knowledge and skills to assess and treat specific child psychiatric conditions.

Financial, legal, and societal aspects	Knowledge of specific CAP related aspects of financial, legal and organization of social and educational system.

Attitudes	Attitudes related to vulnerability of children and parents, safeguarding and stigma.

*Phase 2a: Developing a prototype of courses for CAP training: guiding principles*	

Workplace learning	the content of cursory education is in line with practice and workplace learning as much as possible

In-depth learning	the curriculum allows for gradually more specialization within CAP with more abstract and overarching themes

Group learning	balance between exchange and cross-pollination between trainees within and between different training groups and regions with an eye for emotional safety and group formation

Faculty development	involvement of teachers and experts in the development of modules at a national level to assure sufficient level of expertise; increase the exchange of teaching methods and content between teachers

Embrace regional and local diversity	define national chalk lines so that every psychiatrist registering as CAP has the same basis; leave enough room for autonomy of regions and teachers (balance around 80/20)

Active and blended learning	trainees prepare themselves to use principles of problem-based learning before following the modules; modules contain as many active learning methods as possible; they can be followed on site as well as online

**PHASE 2B: DEVELOPING A PROTOTYPE OF COURSES FOR CAP TRAINING: LAY-OUT OF MODULES**	

**Module characteristics**	**Module title example and covered DSM-classification(s)**

Specific conditions 10 modules 16 hours (4 half days) each Blended learning. Organized at regional level. Online participation of trainees from other regions allows alignment with workplace learning, flexible personal schedules. Online participation of experts from other regions.	Controlling behavior and focusing attention (Attention deficit and hyperactivity disorders)

	A healthy mind in a healthy body (Sleeping disorders, eating disorders, neurological conditions)

	Too much of a good thing (Addiction and substance abuse disorders)



General skills 6 modules 8 hours (1 day) each In person learning. Organized at national level. Live participation allows for group building and network activities for future CAP workforce. Interaction with national experts serves professional development.	Roles of CAP in specialist mental health care and advocacy

	Laws, regulations, and social aspects of CAP

	Genetics of CAP conditions and phenotypical traits

	Neurobiology and new treatments

	Cooperation in networks with other professionals

	Lifelong learning and development and teaching as a CAP


### Second phase: Developing a prototype of courses for CAP training

The vision was translated into guiding principles that could be used for theoretical course development (see Table 1, phase 2a). Next, a decision was made to develop a program of courses covering the list of learning objectives (see Table 1 for titles and content, phase 2b) at a national level and to organize them at a regional level with access for all. Overall teaching time of these 16 modules was about 30% less compared to previous theoretical courses to allow time for more personal projects towards the end of training.

### Third phase: Pilots of courses

A core group led by a course-coordinator set out to develop a course prototype. They started with a preliminary online wall based on the learning goals from phase 1 and teaching methods guided by principles from phase 2. It contained sections with previously used materials, learning objectives, a preliminary program for the new module and literature, assignments, presentations, video lectures, and podcasts.

This first version was provided to a regional group of trainees within their regular training course program. Most core group members participated actively in the teaching, even when it was provided in another region, making use of blended methods (e.g., pre-recorded videos or live sessions with online teachers). The input from these trainers was used for evaluation purposes. Experiences from trainees (around n = 10 each time a course was provided) and trainers about the strengths and weaknesses of the course were collected at the end of the course by the course-coordinator. The online walls were open source for trainees and teachers allowing a consecutive number of pilots with direct feedback from all stakeholders and simultaneous use for teaching.

### Fourth phase: Evaluation, redesign, and further implementation

Every three months, national CAP trainers convened to update and engage additional teaching staff, aligning with their interests to motivate participation and time reallocation. When a course was provided in one of the regions, feedback was collected via the online media wall, facilitating iterative cycles and alignment with common training goals.

Considering the actual start of a full cycle at a national level in the Netherlands, the day of the week teaching was provided needed to be aligned. Although this may seem mundane, it required substantial changes in local hospitals and training centers. In line with what has been described in the *Carpe Diem* methodology, alignment of common goals and learning objectives helped reallocate and re-organize regional resources in favor of cooperation at a national level.

The online media-walls for all modules were continuously and simultaneously available for all teachers and trainees at a national level throughout the process. Thus, courses were accessible for online participants and experts could participate as guest-speakers across regions. Teacher and trainee online feedback on the media-wall helped to improve courses in the short term and throughout the next iterative cycles. An exception to blended learning was made for the 6 day-long national modules that were organized at a central location for all trainees to allow for group formation and networking.

All in all, this cyclical national program integrated knowledge from national experts in the field of CAP into theoretical courses, offered personalized training schedules and allowed general psychiatrists to sub-specialize in CAP aligned with their continuous medical educational learning needs.

## Critical reflection

The four phases of the flexible instructional design method took place in a flexible manner ‘while living on site’, together with a wide stakeholder’s group, supported by technology, and within the reality of CAP as a smaller medical sub-specialty with limited resources. Once common vision and learning goals were aligned with changes in the field of CAP and the prototype was developed (phases 1 and 2), the first courses could be tested in daily practice (phase 3). Phase 4 refined these courses in brief iterative cycles and allowed for designing additional courses building on and adding to previous experiences in brief iterative cycles. Although other methods of educational re-design – with some adjustment – could also provide flexible re-design, the lessons learned with the application of the Carpe Diem method are worth reflecting on to provide insight to others hoping to adopt a similar process.

1. Starting with a common vision and learning objectives in line with new insights in the field of a medical specialty within a broad stakeholder’s group creates common trust that allows testing prototypes in brief iterative cycles.
2. Working with online open-source media-walls helps to support a balanced blended learning approach and resolves logistical challenges to fit theoretical modules with personal training and education schedules.
3. Re-designing courses at a national level requires an initial investment from clinician-educators but when weaved into daily practice readily saves them time.
4. Setting up a flexible re-design method pays back in the short term and holds promise to easily adapt courses to future changes in the field.
5. Specifically for CAP, the flexible growth mindset and collaborative approach of the Carpe Diem method resonates well with developmental and systems notions that are key.

To assess and evaluate the value of our process for other post-graduate training settings, a commentary perspective was collected from international CAP educational specialists and from pediatric post-graduate scholars from the Netherlands. After implementation, the project was presented to an international group of 24 educational experts in CAP. One of the pilot courses was translated into English using an automatic online translation tool and presented. The international experts were positive about the open-source method as it avoids scattering of materials in folders and e-mails and it is simultaneous availability for teachers and trainees. The methods were judged as valuable and with slight adaptions useful in other (inter-) national settings. Appreciation was expressed for the interactive and collaborative way of development. It was expected that trainees would gain from a better fit with challenging personal schedules and teachers from workload reduction and inspiration. Points of concern regarding implementation of the method in their home countries included safe-guarding intellectual property rights and reimbursement for module coordinators, trainees’ privacy, and IT back-ups. Respecting cultural differences, countries would prefer to copy the module and modify it for their own country or region.

Three post-graduate experts in pediatrics as a related medical post-graduate training program expected that pediatric subspecialties (e.g., pediatric intensive care, neonatology, neonatal neurology, …) are coping with similar challenges. Aspects of the presented redesign deemed relevant included a central and attractive digital access point, accommodation of highly individualized learning tracks, and agreement on and adherence to a national curriculum as a basis for joint theoretical course development. As challenges, they noted finding a balance between transferable curriculum items while recognizing the importance of local context in teaching courses.

## Conclusions

The development of high-quality teaching modules is feasible, even for (sub-) specialties with relatively small numbers of trainees and trainers. In other words, it is possible to rebuild the house while living on site. Using a flexible iterative re-design model, facilitated by technology offers additional advantages to the specialty at a national and international level without an increased need for additional resources and time. A longer-term implementation evaluation is needed to show to what extent the investment made in the re-design proves to be future-proof and helps adjust to future changes in the field.
